# Endovascular Aortic Repair of an Infective Native Aortic Aneurysm

**DOI:** 10.7759/cureus.63988

**Published:** 2024-07-06

**Authors:** Taishi Fujii, Kai Machida, Sakamoto Daisuke, Nagayoshi Yasuhiro, Tamaki Takano

**Affiliations:** 1 Department of Cardiothoracic Surgery, Kanazawa Medical University, Uchinada, JPN; 2 Department of Cardiovascular Surgery, Kanazawa Medical University, Uchinada, JPN

**Keywords:** blood culture, treatment of infective native aortic aneurysm, antibiotic therapy, mycotic aortic aneurysm, endovascular aortic repair, infective native aortic aneurysm

## Abstract

An infective native aortic aneurysm (INAA) is a rare, life-threatening, and complex disease. Therefore, the diagnosis and treatment of INAA remain uncertain. We describe the case of a 64-year-old man who had abdominal pain and a fever for more than one week. We diagnosed him with INAA on the basis of the clinical presentation, laboratory findings, and computed tomography (CT) images. After administering preoperative antibiotic therapy for four weeks, we performed endovascular aortic repair (EVAR). He then received antibiotic treatment for 12 months postoperatively. After successful treatment of an INAA with endovascular aortic repair, the patient had no recurrence for more than six years after the end of antibiotic therapy.

## Introduction

Infective native aortic aneurysm (INAA) is a rare, life-threatening, and complex disease that accounts for 0.65-2.0% of all aortic aneurysms in European countries and the USA, and it has a prevalence of 13% in Taiwan [1]. Although the optimal methods for the diagnosis and treatment of INAA remain uncertain, the conventional treatment is aneurysm resection with tissue debridement and revascularization, followed by long-term antibiotic therapy. However, the morbidity and mortality rates after surgical management are high [2-4]. Recently, endovascular aortic repair (EVAR) has become widely accepted as a minimally invasive procedure with similar mortality and morbidity rates to open surgery in treating INAA [1,4].

There is no consensus on the optimal antimicrobial regimen for INAA. However, postoperative antibiotic therapy is recommended for at least six weeks and as long as possible, depending on the individual circumstances.

We describe a case of INAA that was successfully treated with EVAR and 12 months of postoperative antibiotic therapy.

## Case presentation

A 64-year-old man presented with abdominal and back pain and a fever (more than 38 °C) for more than eight days. His blood pressure was 159/90 mmHg on admission. A physical examination revealed a pulsatile mass in the left abdomen. Computed tomography (CT) confirmed the presence of an abdominal aneurysm that had periaortic fat stranding and a periaortic soft-tissue mass (Video 1).

**Video 1:** Enhanced CT at admission Computed tomography at admission shows an abdominal aneurysm with periaortic fat stranding and a periaortic soft-tissue mass.

Routine blood testing revealed that the peripheral white blood cell count was 12.5 × 10^9^/L, and the C-reactive protein concentration was 22.5 mg/L. A transthoracic echocardiogram revealed no endocarditis or vegetation. We suspected INAA on the basis of the clinical manifestations, physical signs, biochemical test results, and CT features.

To treat this persistent infection, we administered the antibiotic meropenem until the blood culture results were available. The antibiotic was then changed to penicillin G for the pathogen *Streptococcus agalactiae*. After four weeks of antibiotic treatment, the infectious findings improved: he had no fever and no abdominal pain, the peripheral white blood cell count was 7.41 × 10^9^/L, and the C-reactive protein concentration was 1.59 mg/L. However, the aneurysm had increased by 13 mm (Video 2), so we determined that surgical treatment was necessary.

**Video 2:** CT after four weeks of antibiotic treatment After four weeks of antibiotic treatment, the aneurysm has expanded by 13 mm.

Since we believed the infection to be under control, we performed EVAR. Abdominal aortic angiography revealed that the aneurysm measured 53 mm × 53 mm and was located in the infrarenal aorta. A Gore Excluder (main body 26 mm × 14.5 mm × 140 mm; W. L. Gore ＆ Associates, Flagstaff, AZ, USA) was passed from the abdominal aorta to the right terminal common iliac artery. A Gore Excluder iliac branch (16 mm × 12 mm × 100 mm) was placed in the contralateral common iliac artery. There were no endoleaks seen on postoperative angiography (Video 3).

**Video 3:** Angiography of endovascular aortic repair (EVAR) Angiography performed after endovascular aortic repair shows no endoleaks.

After the operation, we administered penicillin G for four weeks, during which time he had a normal temperature, no abdominal pain, and normal blood test results. Postoperative CT revealed satisfactory isolation of the aneurysm (Video 4).

**Video 4:** CT: four weeks postoperatively Computed tomography performed four weeks postoperatively shows that the stent-graft isolated the native blood flow from the infective aneurysm.

The antibiotic therapy was then changed to 250 mg of oral amoxicillin three times daily. As a precautionary measure, we decided to treat the patient with antibiotics for 12 months postoperatively. He was discharged 31 days after the surgery. One year after the surgery, the antibiotic therapy was discontinued. It has been six years since the end of the antibiotic therapy. There has been no recurrence, and the aortic aneurysm has almost disappeared (Video 5).

**Video 5:** CT: six years postoperatively At six years postoperatively, there are no signs of infection on computed tomography images.

## Discussion

INAA is an aortic aneurysm that occurs secondary to infection and has a high morbidity rate and a reported mortality rate of 21% to 36% [2-4]. INAA is an uncommon condition, and its management remains a challenging clinical problem. Until recently, there had been no consensus regarding INAA diagnostic criteria and standards. However, the European Society for Vascular Surgery recently created guidelines that recommended INAA diagnosis based on a combination of clinical presentation, laboratory findings, and imaging [3]. Additionally, a diagnostic algorithm for INAA has been reported [5].

Patients with INAA are often older adults with concomitant infections or sepsis and cardiovascular comorbidities [1]. Furthermore, 75% of patients with INAA are men [1,2]. The typical symptoms of INAA at presentation are pain and fever. We diagnosed our patient with INAA on the basis of the presence of abdominal pain, fever, positive bacterial culture, and CT findings.

The blood culture results of patients with INAA are often negative. The rate of positive blood culture has been reported as 44.4% in a Japanese study but ranges from 40% to 80% in other studies [6]. Studies from Asian countries such as Taiwan, Thailand, and Singapore have reported that the major pathogens involved in INAA are Salmonella species, which account for 50-80% of cases [6]. In contrast, studies from European countries have reported that the most frequent pathogens involved in INAA are Gram-positive cocci, which account for 30-60% of cases. The Japanese study reported that Gram-positive cocci and Gram-negative rods each accounted for almost half of all patients with INAA [6]. INAAs caused by Salmonella species show rapid disease progression and carry a risk of early rupture [7]. Sörelius et al. reported that after the early postoperative period with high mortality, the long-term prognosis of Salmonella-positive patients was favorable, while non-Salmonella-positive patients might have serious late complications [7]. In our case, the patient was Japanese, and his blood culture revealed the presence of S. agalactiae.

The gold standard treatment for INAA is resection of the aneurysm, debridement of the infected aorta and the surrounding tissues, the use of muscle flaps or the great omentum to cover the infected field, and either an in situ or extra-anatomical reconstruction followed by long-term antibiotic therapy [1,7-9]. However, recent reports indicate that there are no differences in survival between open surgery and EVAR for the treatment of INAA [1,4,6-12]. We decided to use EVAR to treat our patient and obtained favorable results.

There is currently no consensus on the optimal antimicrobial regimen for INAA. The current recommendation is to initiate empirical antibiotic therapy with agents effective against Gram-positive cocci and Gram-negative rods in cases of suspected INAA after securing a culture specimen. The American Heart Association guideline recommends at least six weeks of postoperative antimicrobial therapy and possibly longer [13]. The European Society of Vascular Surgery guideline recommends that the duration of antibiotic therapy range from four to six weeks to lifelong and should be decided on a case-by-case basis [14]. The reported postoperative antibiotic treatment strategies for INAA vary from absent to 2-6 weeks, 3-6 months, 6-12 months, and even lifelong [3,4,13,14]. However, long-term antibiotic therapy for more than one month can cause multidrug-resistant bacterial infections [15]. In our case, we ended the antibiotic therapy after 12 months because we judged that the infection was controlled on the basis of the blood examination results and CT findings. Six years have passed since the end of antibiotic treatment, and there have been no signs of infection in that time.

## Conclusions

We performed EVAR to treat INAA and then administered 12 months of postoperative oral antibiotic treatment. At the time of writing this article, there had been no recurrence or complications. We consider that postoperative antibiotic therapy should be selected on an individual case-by-case basis and should be finished when the infection is controlled, as long-term antibiotic treatment could result in multidrug-resistant bacterial infection. However, the duration of antibiotic therapy after surgical INAA treatment needs to be further evaluated through long-term follow-up.
